# Deletion of *Alox15* improves kidney dysfunction and inhibits fibrosis by increased PGD_2_ in the kidney

**DOI:** 10.1007/s10157-021-02021-y

**Published:** 2021-02-17

**Authors:** Naohiro Takahashi, Hiroaki Kikuchi, Ayaka Usui, Taisuke Furusho, Takuya Fujimaru, Tamami Fujiki, Tomoki Yanagi, Yoshiaki Matsuura, Kenichi Asano, Kouhei Yamamoto, Fumiaki Ando, Koichiro Susa, Shintaro Mandai, Takayasu Mori, Tatemitsu Rai, Shinichi Uchida, Makoto Arita, Eisei Sohara

**Affiliations:** 1 Department of Nephrology, Tokyo Medical and Dental University (TMDU), 1-5-45 Yushima, Bunkyo-ku, Tokyo, 113-8510 Japan; 2 Laboratory of Immune Regulation, The School of Life Sciences, Tokyo University of Pharmacy and Life Sciences, Tokyo, Japan; 3 Department of Comprehensive Pathology, Graduate School of Medical and Dental Sciences, Tokyo Medical and Dental University (TMDU), Tokyo, Japan; 4 Laboratory for Metabolomics, RIKEN Center for Integrative Medical Sciences (IMS), 1-7-22 Suehiro-cho, Tsurumi-ku, Yokohama City, Kanagawa 230-0045 Japan; 5 Division of Physiological Chemistry and Metabolism, Graduate School of Pharmaceutical Sciences, Keio University, Tokyo, Japan

**Keywords:** Chronic kidney disease, Lipoxygenase, ALOX15, Mediator lipidomics, Polyunsaturated fatty acids, Fibrosis

## Abstract

**Background:**

Lipid-metabolizing enzymes and their metabolites affect inflammation and fibrosis, but their roles in chronic kidney disease (CKD) have not been completely understood.

**Methods:**

To clarify their role in CKD, we measured the mRNA levels of major lipid-metabolizing enzymes in 5/6 nephrectomized (Nx) kidneys of C57BL/6 J mice. Mediator lipidomics was performed to reveal lipid profiles of CKD kidneys.

**Results:**

In 5/6 Nx kidneys, both mRNA and protein levels of *Alox15* were higher when compared with those in sham kidneys. With respect to in situ hybridization, the mRNA level of *Alox15* was higher in renal tubules of 5/6 Nx kidneys. To examine the role of *Alox15* in CKD pathogenesis, we performed 5/6 Nx on *Alox15*^−/−^ mice. *Alox15*^−/−^ CKD mice exhibited better renal functions than wild-type mice. Interstitial fibrosis was also inhibited in *Alox15*^−/−^ CKD mice. Mediator lipidomics revealed that *Alox15*^−/−^ CKD mouse kidneys had significantly higher levels of PGD_2_ than the control. To investigate the effects of PGD_2_ on renal fibrosis, we administered PGD_2_ to TGF-β1-stimulated NRK-52E cells and HK-2 cells, which lead to a dose-dependent suppression of type I collagen and αSMA in both cell lines.

**Conclusion:**

Increased PGD_2_ in *Alox15*^−/−^ CKD mouse kidneys could inhibit fibrosis, thereby resulting in CKD improvement. Thus, *Alox15* inhibition and PGD_*2*_ administration may be novel therapeutic targets for CKD.

**Supplementary Information:**

The online version contains supplementary material available at 10.1007/s10157-021-02021-y.

## Introduction

Polyunsaturated fatty acids (PUFA) and their metabolites are linked to inflammation and its resolution in several organs [1]. Oxylipins, which are produced by the oxidation of PUFA, are important for PUFA biological activity as lipid mediators [1, 2]. Biosynthesis of oxylipins is mediated by several enzymes, such as lipoxygenase (LOX), cyclooxygenase (COX) and cytochrome P450 (CYP) [3]. These enzymes produce several lipid metabolites, such as prostaglandins, leukotrienes, and lipoxins, which are all heavily involved in the regulation of inflammation [1, 4]. For instance, ALOX15, a major subtype of LOX, has a dual aspect of proinflammatory and anti-inflammatory properties through its metabolites [5]: ALOX15 is highly expressed in eosinophils, bronchoalveolar epithelial cells and alveolar macrophages under nonpathological condition [5, 6], and promotes severity of asthma [7], lung injury [8], and heart failure [9], whereas it counteracts inflammation in arthritis [10] and ischemic brain [11]. Additionally, lipid mediators and their enzymes affect organ fibrosis as well as inflammation. Specific lipid mediators are involved in the pathogenesis of lung [12], liver [13], and heart [14] fibrosis. Similarly, the above-mentioned ALOX15 is also linked to the pathogenesis of fibrosis such as dermal fibrosis [15].

One of the common chronic diseases is chronic kidney disease (CKD), which affects approximately 8–16% of the general population in all stages combined [16]. Although CKD pathogenesis is complex and varies depending on the underlying disease, the kidney tissue generally becomes dysfunctional, leading to end-stage renal failure caused by chronic inflammation and subsequent fibrosis [17]. As mentioned, lipid mediators derived from PUFAs are strongly linked to inflammation and its resulting fibrosis. For instance, lipoxins and resolvins inhibit renal fibrosis [18, 19], but these effects are not yet examined in the CKD model with impaired kidney function. Moreover, comprehensive lipidomic profiles in CKD kidney tissues are still unreported. Thus, the role of lipid metabolic enzymes and their products in CKD pathogenesis has remained poorly understood.

This study aimed to elucidate the involvement of fatty acid metabolizing enzymes and their products in the renal impairment of 5/6 nephrectomized (Nx) CKD model mice. This study revealed that both of the transcription and protein expression levels of *Alox15* were increased in CKD kidneys, and *Alox15*^−/−^ mice demonstrated improved kidney dysfunction and fibrosis in the CKD model. Moreover, PGD_2_, which is the increased lipid metabolite in the CKD kidneys of *Alox15*^−/−^ mice, inhibited the epithelial–mesenchymal transition (EMT) in proximal tubular cultured cells. Therefore, *Alox15* inhibition and/or PGD_2_ administration could be a novel therapeutic target of CKD and fibrosis.

## Materials and methods

### Animals and experiments

This study used 8-week-old male C57BL/6 J mice (CLEA Japan), which were acclimatized for 1 week before all the experiments were performed. Moreover, *Alox15*^−/−^ mice were generated in the C57BL/6 J background (Jackson Laboratory), and the 5/6 Nx model was established according to a previous study [20]. We collected blood samples for the evaluation of renal function and kidney tissue samples for immunoblotting and polymerase chain reaction at 8 weeks after 5/6 Nx, and for histological analysis at 40 weeks after 5/6 Nx. All experiments conformed to the guidelines for animal research of TMDU, and The Animal Care and Use Committee of TMDU approved our study protocol (approval number: A2019-117C4).

### LC–MS/MS-based mediator lipidomics

We conducted LC–MS/MS analysis as described previously [21, 22]. Details of the procedure are described in Supplementary Methods.

### Other experimental methods

We described other experimental methods in Supplementary Methods.

## Results

### mRNA level of *Alox15* was increased in 5/6 Nx kidney

**Fig. 1:**
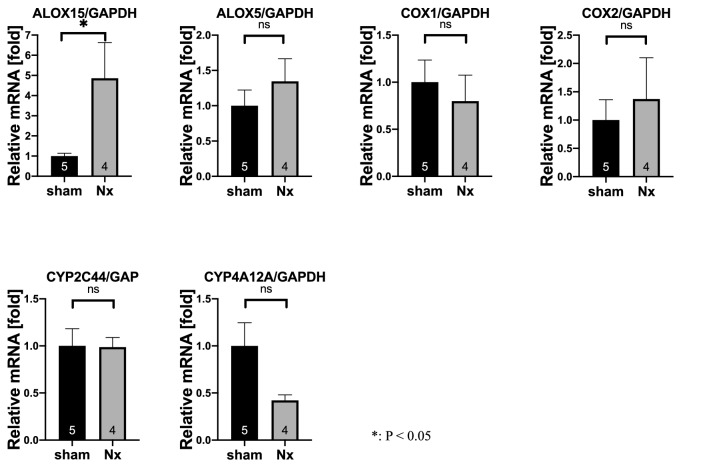
mRNA levels of major renal oxylipin enzymes in CKD kidney samples. Quantitative reverse transcription-polymerase chain reaction (qRT-PCR) analysis of major oxylipin enzymes expressed in the kidney (*Alox15*, *Alox5*, *Ptgs1 [COX1]*, *Ptgs2 [COX2], Cyp4a12* and *Cyp2c44*) was performed using sham and 5/6 nephrectomized (Nx) kidney samples. Compared with the sham kidneys, the 5/6 Nx kidneys had significantly increased mRNA level of *Alox15* (*P* = 0.0004). The number of samples is shown at the bottom of the bar graph. Values are mean ± SEM. Unpaired Student’s *t* test, *P < 0.05

The quantitative changes of oxylipin enzymes in the CKD kidney were investigated by applying C57BL/6 mice to sham operation or 5/6 Nx as described previously [20]. To analyze the expression of major oxylipin enzymes expressed in the kidney (*Alox15*, *Alox5*, *Ptgs1 (COX1)*, *Ptgs2 (COX2), Cyp4a12* and *Cyp2c44*) [23, 24], we extracted kidney samples. Among the enzymes, *Alox15* in 5/6 Nx kidneys had a significantly elevated mRNA level (*P* = 0.0004) compared with that in sham kidneys (Fig. 1). Conversely, the mRNA levels of the other major enzymes did not significantly change in this CKD model.

### Protein level of *Alox15* was increased in renal proximal tubular cells

**Fig. 2:**
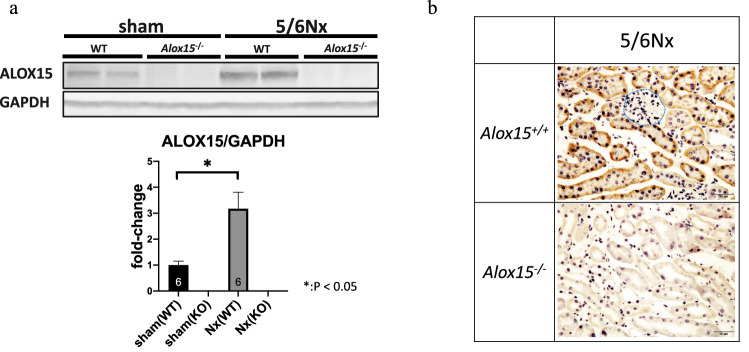
*Alox15* expression in 5/6 Nx kidney at the protein level and localization of the mRNA of *Alox15* in kidney tissues. **a** (upper) Representative immunoblots of ALOX15 of kidneys from sham-control and 5/6 nephrectomized (Nx) CKD mice. The band of ALOX15 was confirmed by the absence of ALOX15 band in *Alox15*^−/−^ mice samples. (lower) Densitometry analysis of immunoblots. The ALOX15 in 5/6 Nx mouse kidneys was significantly increased compared with sham-control mouse kidneys (*P* = 0.0078). The number of samples is given at the bottom of the bar graph. Values are mean ± SEM. Unpaired Student’s *t* test, *P < 0.05. **b** in situ hybridization of kidneys from 5/6 Nx *Alox15*^+*/*+^ mice and 5/6 Nx *Alox15*^−/−^ mice. In 5/6 Nx mouse kidneys, *Alox15* mRNA was strongly expressed in the tubules, especially proximal tubules. The glomerulus had no mRNA expression of *Alox15* (the blue border indicates a glomerulus)

In 5/6 Nx kidneys, the ALOX15 protein levels were also increased (*P* = 0.0078), as expected by increased transcriptional levels (Fig. 2a). To determine which cell types had increased Alox15 protein level in the CKD kidney, we examined the localization of *Alox15* mRNA by in situ hybridization. In situ hybridization to mRNA revealed that the high expression level of *Alox15* was localized at renal tubular cells in the 5/6 Nx model, but not in glomeruli (Fig. 2b). Histological features suggested that *Alox15* mRNA was strongly expressed in the proximal tubules.

### *Alox15*^*−/−*^ mice were resistant to renal damage and fibrosis in the 5/6 Nx model

**Fig. 3:**
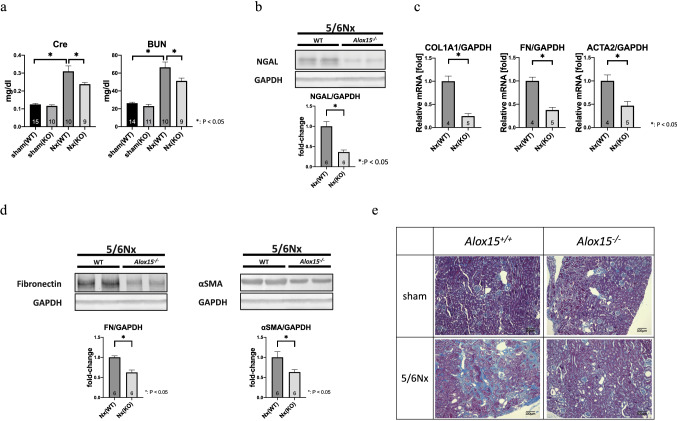
*Alox15*^−/−^ mice demonstrated kidney function and renal fibrosis amelioration in the CKD model. **a** Serological data from CKD mice at 8 weeks after 5/6 nephrectomy (Nx) showed that both serum creatinine and blood urea nitrogen levels of *Alox15*^−/−^ CKD mice were significantly lower than those of WT CKD mice. The number of samples is shown at the bottom of the bar graph. Values are mean ± SEM. One-way analysis of variance was followed by Tukey’s multiple comparisons test, *P < 0.05. **b** Representative immunoblots of NGAL (a renal damage marker) of kidneys from 5/6 Nx mice. NGAL was suppressed in *Alox15*^−/−^ CKD mouse kidneys compared with that in WT CKD mice. The number of samples is shown at the bottom of the bar graph. Values are mean ± SEM. Unpaired Student’s *t* test, *P < 0.05. **c** The mRNA of collagen type I and fibronectin (fibrosis markers), and *Acta2* (αSMA; an EMT marker) were significantly suppressed in *Alox15*^−/−^ mouse kidneys under 5/6 Nx condition compared with WT CKD mouse kidneys. The number of samples is shown at the bottom of the bar graph. Values are mean ± SEM. Unpaired Student’s *t* test, *P < 0.05. **d** Representative immunoblots of fibronectin and αSMA of kidneys from 5/6 Nx mice. Both of fibronectin and αSMA were suppressed in *Alox15*^−/−^ CKD mouse kidneys compared with that in WT CKD mice. The number of samples is shown at the bottom of the bar graph. Values are mean ± SEM. Unpaired Student’s *t* test, *P < 0.05. **e** Kidney with Masson’s trichrome staining. Kidneys from *Alox15*^+*/*+^ and *Alox15*^−/−^ mice under sham and 5/6 Nx conditions, respectively. Kidney tissues were obtained at 40 weeks after 5/6 Nx. Although interstitial fibrotic changes are evident in 5/6 Nx mouse kidneys, interstitial fibrosis in 5/6 Nx *Alox15*^−/−^ mouse kidneys was significantly improved compared with that in 5/6 Nx *Alox15*^+*/*+^ mouse kidneys

In 5/6 Nx CKD kidneys, the transcriptional and protein expression levels of ALOX15 were elevated. Therefore, we investigated whether and how ALOX15 plays a role in CKD in terms of kidney dysfunction and renal fibrosis. For this purpose, *Alox15*^−/−^ mice were applied to the 5/6 Nx CKD model. Interestingly, serum Cre and blood urea nitrogen (BUN) levels were lower in *Alox15*^−/−^ 5/6 Nx mice than those in wild-type (WT) mice (Fig. 3a), indicating that *Alox15* depletion showed a protective effect in CKD pathogenesis. Accordingly, NGAL (a renal damage marker) protein expression was suppressed in *Alox15*^−/−^ mice compared with that in WT mice (Fig. 3b). In addition, *Alox15*^−/−^ mouse kidneys showed reduced *Col1a1, Fn* and *Acta2* (αSMA) mRNA levels (Fig. 3c), and also showed decreased fibronectin and αSMA protein expression (Fig. 3d). Moreover, *Alox15*^−/−^ mouse kidneys exhibited clearly suppressed interstitial fibrotic changes shown in Masson’s trichrome staining (Fig. 3e). Therefore, *Alox15* deletion ameliorates kidney dysfunction and fibrosis in the CKD animal model.

### Mediator lipidomics revealed altered PUFA metabolism in *Alox15*^*−/−*^ CKD kidneys

**Fig. 4:**
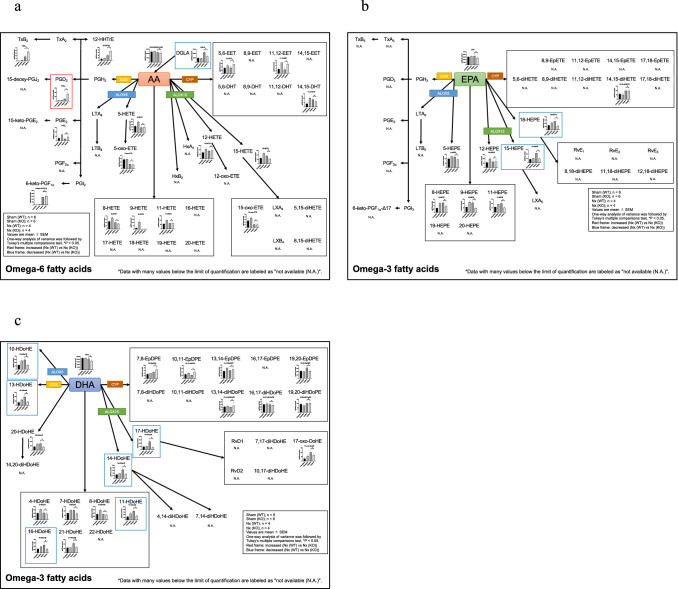
Pathway maps of lipid mediators derived from omega-6 and omega-3 fatty acids. **a** Pathway map of lipid mediators derived from arachidonic acids (AA). **b** Pathway map of lipid mediators derived from eicosapentaenoic acids (EPA). **c** Pathway map of lipid mediators derived from docosahexaenoic acids (DHA)

**Fig. 5:**
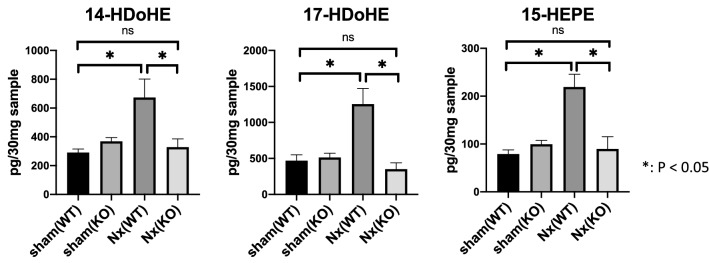
Lipidomics confirmed that the production of ALOX15-dependent lipid metabolites was suppressed in *Alox15*^*−/−*^ CKD kidneys. 14-HDoHE, 17-HDoHE, and 15-HEPE, which are ALOX15-dependent lipid metabolites, were significantly increased in WT CKD kidneys (*P* = 0.0018, 0.0008, < 0.0001 respectively). The increase was suppressed in *Alox15*^*−/−*^ mice. The number of samples is as follows: sham (WT), *n* = 6, sham (KO), *n* = 6, Nx (WT), *n* = 4, Nx (KO), *n* = 4. Values are mean ± SEM. One-way analysis of variance was followed by Tukey’s multiple comparisons test, *P < 0.05

**Fig. 6:**
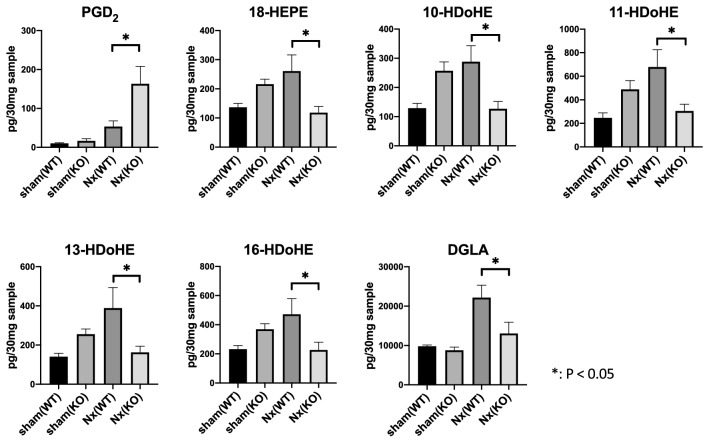
Lipid metabolites which were significantly different between *Alox15*^+*/*+^ and *Alox15*^*−/−*^ mice under 5/6 Nx condition. *Alox15*^−/−^ CKD kidneys had significantly increased levels of PGD_2_ compared with WT CKD kidneys (*P* = 0.0093). In contrast, *Alox15*^−/−^ CKD kidneys had significantly decreased levels of 18-HEPE, 10-HDoHE, 11-HDoHE, 13-HDoHE, 16-HDoHE and DGLA compared with WT CKD kidneys (*P* = 0.0186, 0.0246, 0.0443, 0.0341, 0.0497, 0.0195 respectively). Additionally, 30 mg of each sample was analyzed with lipidomics. Sham (WT); *n* = 6, sham (KO); *n* = 6, Nx (WT); *n* = 4, Nx (KO); *n* = 4. Values are mean ± SEM. One-way analysis of variance was followed by Tukey’s multiple comparisons test, *P < 0.05

As mentioned in the Introduction section of this paper, the biological effects of *Alox15* are related with the lipid mediators generated by *Alox15*. To elucidate the profile of lipid metabolites produced by *Alox15* in the CKD kidney, we examined and compared the sham and 5/6 Nx kidney samples by LC–MS/MS-based mediator lipidomics to determine the lipid mediator profiles between WT mice and *Alox15*^−/−^ CKD mice (Supplementary Table 1). Table 1 shows lipid metabolites which were significantly different between *Alox15*^+*/*+^ and *Alox15*^−/−^ mice under 5/6 Nx condition. In addition, we made pathway maps of lipid mediators with classification by their substrates and their metabolizing enzymes (Fig. 4). A series of *Alox15*-derived PUFA metabolites such as 14-HDoHE, 17-HDoHE, and 15-HEPE [5, 25–27] was significantly increased in CKD models that correlated well to the increased level of ALOX15 expression in the kidneys (*P* = 0.0018, 0.0008, < 0.0001, respectively), and their elevations under CKD conditions were completely suppressed in *Alox15*^−/−^ mice (Fig. 5). Besides 14-HDoHE, 17-HDoHE, and 15-HEPE, the levels of 18-HEPE, 10-HDoHE, 11-HDoHE, 13-HDoHE, 16-HDoHE and DGLA were also significantly decreased in *Alox15*^−/−^ CKD kidneys compared to those in WT CKD kidneys, whereas only PGD_2_ was significantly increased in *Alox15*^−/−^ CKD kidneys (Fig. 6).

**Table 1:** List of lipid metabolites which were significantly different between *Alox15*^+*/*+^ and *Alox15*^*−/−*^ mice under 5/6 Nx condition

Sample name	Sham WT (*n* = 6)		Sham KO (*n* = 6)		Nx WT (*n* = 4)		Nx KO (*n* = 4)		*P* value (Nx WT vs Nx KO)	Increased or decreased in KO compared to WT
	Ave	SE	Ave	SE	Ave	SE	Ave	SE		
PGD2	10.0	1.5	16.5	5.9	53.4	14.5	163.1	45.1	0.0093	Increased
15-HEPE	78.8	8.7	99.4	8.1	219.2	26.7	89.5	25.9	0.0006	Decreased
18-HEPE	136.9	13.3	215.7	17.6	260.8	55.7	118.3	21.7	0.0186	Decreased
10-HDoHE	128.8	16.5	256.9	30.7	288.1	54.7	126.7	25.4	0.0246	Decreased
11-HDoHE	245.9	43.2	488.0	74.5	678.4	146.6	305.5	57.8	0.0443	Decreased
13-HDoHE	140.8	17.4	255.2	26.8	388.1	104.6	162.8	31.2	0.0341	Decreased
14-HDoHE	289.5	25.1	368.6	26.2	673.2	128.3	328.0	57.6	0.0092	Decreased
16-HDoHE	232.1	25.1	369.1	38.3	471.7	106.7	227.0	53.4	0.0497	Decreased
17-HDoHE	468.5	82.0	513.1	59.0	1253.0	218.2	350.6	88.7	0.0005	Decreased
DGLA	9794.6	349.6	8797.8	791.8	22,167.8	3136.0	13,040.0	2866.7	0.0195	Decreased

By mediator lipidomics, the above fatty acid metabolites were detected in the kidney tissue (30 mg). The *P* values in the table were obtained by comparing *Alox15*^+*/*+^ and *Alox15*^*−/−*^ mice under 5/6 Nx condition. The number of samples is as follows: sham (WT), *n* = 6, sham (KO), *n* = 6, Nx (WT), *n* = 4, Nx (KO), *n* = 4. One-way analysis of variance was followed by Tukey’s multiple comparisons test

### PGD_2_ suppressed EMT in cultured kidney cells

**Fig. 7:**
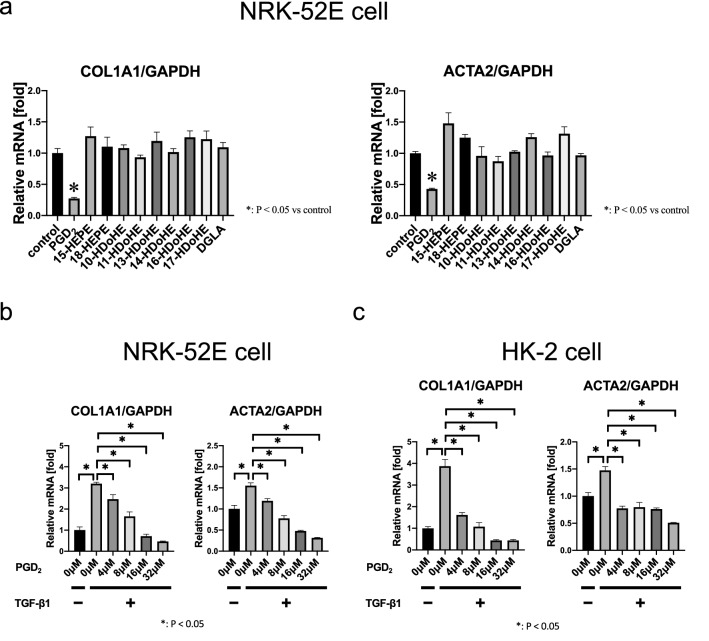
PGD_2_ suppressed EMT in cultured kidney cells. **a** Effects of lipid metabolites which were significantly different between *Alox15*^+*/*+^ and *Alox15*^*−/−*^ mice under 5/6 Nx condition on the EMT of NRK-52E cells. These lipid metabolites were administered at 8 μM each to NRK-52E cells with TGF-β1 (5 ng/mL). PGD_2_ significantly suppressed *Col1a1* and αSMA (*Acta2*) expression. None of the other fatty acid metabolites inhibited or promoted *Col1a1* and αSMA. **b** Effects of PGD_2_ on the EMT of NRK-52E cells. PGD_2_ significantly suppressed *Col1a1* and *Acta2* expression induced with TGF-β1 (5 ng/mL) in NRK-52E cells dose-dependently. **c** Effects of PGD_2_ on the EMT of HK-2 cells. PGD_2_ also suppressed *Col1a1* and *Acta2* expression induced with TGF-β1 (5 ng/mL) in HK-2 cells dose-dependently. *n* = 3 for each group. Values are mean ± SEM. One-way analysis of variance was followed by Tukey’s multiple comparisons test, *P < 0.05

The effects of lipid metabolites which were significantly different between *Alox15*^+*/*+^ and *Alox15*^−/−^ mice under 5/6 Nx condition were examined by administering these lipids to NRK-52E cells that were activated by pro-fibrotic cytokine TGF-β1. We assessed the mRNA expression of *Cola1a* and *Acta2* (αSMA) in response to TGF-β1 to determine the effects of these lipids in culture (Fig. 7a). Among those, PGD_2_ conferred potent antifibrotic effect on NRK-52E cells in response to TGF-β1. The suppressive effect of PGD_2_ on COL1A1 and αSMA expression were dose dependent with the EC50 of 7.12 μM and 6.48 μM, respectively (Fig. 7b). Additionally, we conducted the same experiments in HK-2 cells, which are immortalized proximal tubule epithelial cells from normal adult human kidneys, and found a similar outcome, that is, COL1A1 and αSMA inhibition in a dose-dependent manner, in response to treatment with PGD_2_ (Fig. 7c). Therefore, increased levels of PGD_2_ in *Alox15*^−/−^ CKD kidneys may contribute to the antifibrotic effects in CKD.

### 15-PGDH, a major PGD_2_-metabolizing enzyme, was reduced in *Alox15*^*−/−*^ CKD kidneys

**Fig. 8:**
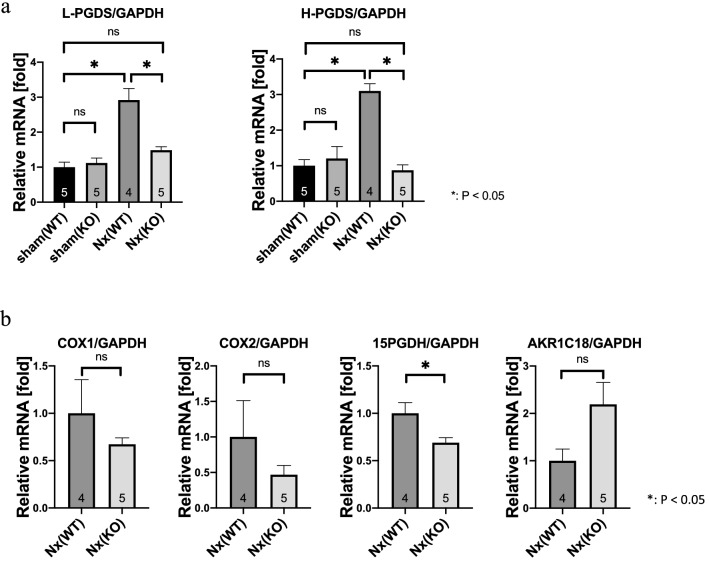
15-PGDH, a major PGD_2_-metabolizing enzyme, was reduced in *Alox15*^*−/−*^ CKD kidneys. **a** Relative mRNA levels of each of the two PGDS synthase isoforms in the kidney tissue of sham or CKD model mice. In WT mice, both L-PGDS and H-PGDS levels were significantly increased under 5/6 Nx conditions when compared with those under sham conditions (both *P* < 0.0001). Conversely, the increase in L-PGDS and H-PGDS under 5/6 Nx conditions was significantly inhibited in *Alox15*^−/−^ mice (*P* = 0.0004, < 0.0001, respectively), and their mRNA levels did not differ from those of sham WT mice. The number of samples is shown at the bottom of the bar graph. Values are mean ± SEM. One-way analysis of variance was followed by Tukey’s multiple comparisons test, *P < 0.05. **b** Relative mRNA levels of PGD_2_-related enzymes in the kidneys of CKD model mice. The mRNA level of 15-PGDH was significantly reduced in CKD kidneys of *Alox15*^*−/−*^ mice when compared with those of WT mice (*P* = 0.0325). The mRNA levels of COX-1, COX-2 and AKR1C18 were not significantly changed between WT mice and *Alox15*^*−/−*^ mice under 5/6 Nx conditions. The number of samples is shown at the bottom of the bar graph. Values are mean ± SEM. Unpaired Student’s *t* test, *P < 0.05

To clarify the mechanism of the increase in PGD_2_ in the kidneys of *Alox15*^*−/−*^ CKD model mice, we measured the mRNA level of PGD_2_ synthase. PGD_2_ synthase (PGDS) has two isoforms: lipocalin PGDS (L-PGDS) and hematopoietic PGDS (H-PGDS) [28]. Both L-PGDS and H-PGDS levels were significantly increased under 5/6 Nx conditions when compared with those under sham conditions in the kidneys of WT mice (Fig. 8a, both *P* < 0.0001) indicating that increased PGD_2_ in CKD is due to increased PGD_2_ synthases. Conversely, the increase in L-PGDS and H-PGDS under 5/6 Nx conditions was significantly inhibited in *Alox15*^−/−^ mice (Fig. 8a) indicating that the increase in PGD_2_ in *Alox15*^−/−^ CKD model mice was not due to increased production by PGDS. Then, we examined mRNA levels of COX-1 and COX-2 as more upstream synthases of the prostaglandin-producing pathway. We also measured mRNA levels of 15-PGDH and AKR1C18, which are known to be major PGD_2_-metabolizing enzymes [29, 30]. While the mRNA levels of COX-1, COX-2, and AKR1C18 were unchanged, the mRNA level of 15-PGDH was significantly reduced in CKD kidneys of *Alox15*^−/−^ mice when compared with those of WT mice (Fig. 8b, *P* = 0.0325), that potentially lead to the increase in PGD_2_ in CKD kidneys of *Alox15*^−/−^ mice.

## Discussion

This study demonstrated that in CKD kidney samples, both of the transcription and protein levels of *Alox15* were increased, and kidney dysfunction and fibrosis were ameliorated in *Alox15*^−/−^ mice. In addition, LC–MS/MS-based mediator lipidomics revealed that *Alox15*^−/−^ CKD mouse kidneys had significantly increased level of PGD_2_ compared with WT mice. PGD_2_ inhibited the EMT of NRK-52E and HK-2 cells; hence, PGD_2_ increase may contribute to the resistance of *Alox15*^−/−^ mice to renal injury and fibrosis.

The relationship between renal disease and lipid profiles using lipidomics on human serum or plasma have been extensively investigated [31–33], but not on kidney tissue. Similarly, although reports on lipidomics using CKD animal models are few [34–36], all of these studies performed lipidomics on plasma samples from a CKD animal model; however, no comprehensive lipidomics on kidney tissues from the CKD model have been reported yet. Direct lipidomics on tissue is also effective, as well as plasma samples, in identifying functional metabolites. Moreover, metabolite changes in tissues, as well as blood samples, are considerably different by organ [20]. In the present study, we thoroughly analyzed the profile of PUFA-derived lipid mediators in the kidney tissues of a CKD animal model.

This study focused on ALOX15, which is one of the major enzymes that metabolize PUFAs. In this study, both protein and mRNA expression levels of ALOX15 were clearly increased in CKD kidneys. We addressed the cell types responsible for the ALOX15 expression in CKD kidneys, and by in situ hybridization, we found an increased expression of ALOX15 mRNA in renal tubular cells in the 5/6 Nx model.

ALOX15 is involved in chronic diseases, such as atherosclerosis, and its deletion in animal disease models improves these diseases [5]. Regarding the association of ALOX15 with renal disease, proteinuria is decreased in *Alox15*^−/−^ mice under glomerular injury by a streptozotocin-induced diabetic nephropathy model [37]. However, this diabetes mellitus mouse model does not show any decline in kidney function; thus, the mechanism on how ALOX15 is associated with impaired kidney function in CKD models has remained unclear. In the present study, using the 5/6 Nx mouse model, we found that ALOX15 deletion ameliorated kidney dysfunction and renal fibrosis in a CKD model.

To date, only IL-4 and IL-13 in monocytes are known to be regulatory factors that directly increase the expression of ALOX15 [5]. However, it remains unclear what increases ALOX15 in renal tubular cells. A variety of cytokines and uremic toxins are known to be increased in the plasma and kidney in CKD [38, 39]. Among them, there might be novel regulators of ALOX15. Further studies are needed to elucidate the major regulatory factors of ALOX15 in CKD.

In our studies to identify specific intervening oxylipins that link *Alox15* deletion to its renoprotective effect in the 5/6 Nx model, we found that PGD_2_ may be involved in the resistance to renal damage caused by ALOX15 deletion. As a general understanding, to generate PGD_2_, COX-1 and COX-2 produce PGG_2_ from arachidonic acids, which is then converted into PGH_2_. When PGH_2_ is metabolized by PGD_2_ synthase (PGDS), PGD_2_ is produced [28]. PGDS has two genetically distinct isoforms, namely, lipocalin-type PGDS (L-PGDS) and hematopoietic-type PGDS (H-PGDS) [28]. Although PGD_2_ was increased in the *Alox15*^−/−^ CKD mouse kidneys, neither L-PGDS nor H-PGDS was increased in the *Alox15*^−/−^ CKD mouse kidneys, despite the large increase in PGDS in the WT CKD mouse kidneys (Fig. 8a). Furthermore, we also revealed that neither COX-1 nor COX-2 was increased in the *Alox15*^−/−^ CKD mouse kidneys (Fig. 8b).

These results suggest that the increase in PGD_2_ in the *Alox15*^−/−^ CKD mouse kidneys was not due to an increase in synthases but due to the increased substrate availability or an inhibition of degradation, and in fact, we have revealed a reduction in 15-PGDH, one of the major PGD_2_ metabolizing enzymes [29], in the *Alox15*^−/−^ CKD mouse kidneys (Fig. 8b). Further investigation is needed to elucidate the reason why the deletion of *Alox15* leads to decrease in 15-PGDH.

As mentioned above, PGD_2_ could be involved in the resistance to renal injury caused by ALOX15 loss. In this study, PGD_2_ inhibited EMT by TGF-β1 in NRK-52E and HK-2 cells, representing proximal tubular cells. PGD_2_ binds to two different G protein-coupled receptors, namely, DP1 and DP2, whose functions are different [28]. However, in our in vitro experiments, PGD_2_ was effective at concentrations ranging from 4 to 32 μM, which are relatively high, considering that the *K*_i_ values of DP1 and DP2 are 1.7 nM and 2.4 nM, respectively [28]. This result indicates that PGD_2_ may exert antifibrotic effects not via G protein-coupled receptors but via other pathways such as PPARγ, through which 15-d-PGJ_2_, a downstream PGD_2_ metabolite, is known to exert its effects [28].

In this study, which focuses on lipid metabolic enzymes and their metabolites, ALOX15 inhibition and/or PGD_2_ administration could be a promising therapeutic target for CKD. Unfortunately, no ALOX15 specific inhibitor that can be used in clinical practice has been developed [40, 41]. Therapeutic targeting of downstream functional metabolites, such as PGD_2_, rather than inhibition of fatty acid metabolizing enzymes, which affect various metabolites, could be a novel ideal CKD therapy.

## Supplementary Information

Below is the link to the electronic supplementary material.

Supplementary file1 (PDF 237 KB)

## Data Availability

All data are available from the corresponding author upon reasonable request.
