# Supplementary material for: Deletion of Alox15 improves kidney dysfunction and inhibits fibrosis by increased PGD2 in the kidney
Source: Clin Exp Nephrol. 2021 Feb 17;25(5):445–55. doi: 10.1007/s10157-021-02021-y (PMC8038997; doi:10.1007/s10157-021-02021-y)
Supplement: Supplementary file 1 — Supplementary file1 (PDF 237 KB) [file 10157_2021_2021_MOESM1_ESM.pdf]

Deletion of *Alox15* improves kidney dysfunction and inhibits fibrosis by increased PGD_2 in the kidney

Naohiro Takahashi, M.D.[1], Hiroaki Kikuchi, M.D., Ph.D.[1], Ayaka Usui[1], Taisuke Furusho, M.D., Ph.D.[1], Takuya Fujimaru, M.D., Ph.D.[1], Tamami Fujiki, M.D.[1], Tomoki Yanagi, M.D.[1], Yoshiaki Matsuura, M.D., Ph.D.[1], Kenichi Asano, M.D., Ph.D.[2], Kouhei Yamamoto, M.D., Ph.D.[3], Fumiaki Ando, M.D., Ph.D.[1], Koichiro Susa, M.D., Ph.D.[1], Shintaro Mandai, M.D., Ph.D.[1], Takayasu Mori, M.D., Ph.D.[1], Tatemitsu Rai, M.D., Ph.D.[1], Shinichi Uchida, M.D., Ph.D.[1], Makoto Arita, Ph.D.[4, 5*], Eisei Sohara, M.D., Ph.D.[1*]

[1] Department of Nephrology, Tokyo Medical and Dental University (TMDU), Tokyo, Japan

[2] Laboratory of Immune Regulation, The School of Life Sciences, Tokyo University of Pharmacy and Life Sciences, Tokyo, Japan

[3] Department of Comprehensive Pathology, Graduate School of Medical and Dental Sciences, Tokyo Medical and Dental University (TMDU), Tokyo, Japan

[4] Laboratory for Metabolomics, RIKEN Center for Integrative Medical Sciences (IMS), Kanagawa, Japan

[5] Division of Physiological Chemistry and Metabolism, Graduate School of Pharmaceutical Sciences, Keio University, Tokyo, Japan

[*] These authors equally contributed to this work.

Supplementary Methods

Cell culture

Rat kidney epithelial cells (NRK-52E cells) were cultured in Dulbecco's modified eagle medium (4.5 g/L glucose) (Nacalai Tesque) supplemented with 5% fetal bovine serum, whereas human renal proximal tubule cells (HK-2 cells) were cultured in DMEM/F12 (Gibco) supplemented with 10% fetal bovine serum. Both NRK 52E cells and HK-2 cells were purchased from ATCC. We seeded these cells on 6-well culture plates to 80% confluence in a complete medium for 24 h and then transferred them to a serum-free medium. Then, they were preincubated with lipid metabolites for 30 min, followed by the treatment of recombinant human TGF-β1 (Pepro Tech) at 5 ng/mL and the lipid metabolites for different dosage as indicated for 24 h. We cultivated all cells at 37 °C under 5% CO_2 condition in a humidified incubator.

Immunoblotting

We extracted protein samples from the kidneys or cultured cells and performed semiquantitative immunoblotting as described previously [1]. We analyzed and quantified the relative intensities of the immunoblot bands through the ImageJ software (National Institutes of Health). We used primary antibodies such as anti-GAPDH (Santa Cruz Biotechnology, #sc32233, 1:1000), anti-ALOX15 (abcam, #ab244205, 1:1000), anti-NGAL (R&D Systems, #AF1857, 1:1000), anti-fibronectin (abcam, #ab2413, 1:1000) and anti-αSMA (abcam, #ab5694, 1:1000) for the experiment. For secondary antibodies, we used the alkaline phosphatase-conjugated anti-rabbit IgG antibody (Promega, #S3738, 1:1000), anti-goat IgG antibody (Promega, #V115A, 1:1000) and anti-mouse IgG antibody (Promega, #S3721, 1:1000).

Quantitative reverse transcription-polymerase chain reaction (qRT-PCR) analysis

Total RNA extracted from the mouse kidneys was reverse-transcribed using ReverTra Ace (TOYOBO), and the qRT-PCR analysis was performed in the Thermal Cycler Dice Real Time System (Takara Bio). The primers and templates were mixed with SYBR Premix Ex Taq II (Takara Bio). Thereafter, the mRNA contents were normalized to GAPDH and then calculated using the comparative CT method. Sequence-specific primers for mice, NRK-52E cells and HK-2 cells are listed below.

Sequence-specific primers for mice

	Species	Forward 5'→3'	Reverse 5'→3'
Gapdh	mouse	CGTGGAGTCTACTGGTGTCTTCAC	CGGAGATGATGACCCTTTTGGC
Alox15	mouse	GAAGACTCTCAAGGCCTGTT	GTCAGAGATACTGGTCGCCG
Alox5	mouse	ACTACATCTACCTCAGCCTCATT	GGTGACATCGTAGGAGTCCAC
Ptgs1	mouse	GGTGCCCTCACCAGTCAATC	ATTCCGAAGCCAGGTCCAGA
Ptgs2	mouse	TGGGGGAAGAAATGTGCCAA	CAGCCATTTCCTTCTCTCCTGT
Cyp4a12a	mouse	GGGACTTCTATCACCTGGAATG	ACTTGGTACAGGAGGGTAGAT
Cyp2c44	mouse	CCCGTTTCTGTCTTCCATCTT	GTCCTGGATCAAACTTCTCTGG
Ptgds	mouse	CGGCCTCAATCTCACCTCTAC	CCTTGGTGCCTCTGCTGAAT
Hpgds	mouse	TCAAGCTGATGCAGTGGTGG	GAAGGCGAGGTGCTTGATGT
Hpgd	mouse	AGCACGGCATCATCGGATTC	GTCCACAAAGCCTGGGCAAA
Akr1c18	mouse	GCCAGGCCATTCTAAGCAAGA	CTCCATGGCCTTCAGAGACAC
Col1a1	mouse	TGACTGGAAGAGCGGAGAGT	GTTCGGGCTGATGTACCAGT
Fn	mouse	AGACTGCAGTGACCACCATTC	AATGTGTCCTTGAGAGCATAG
Acta2	mouse	GCTGCTCCAGCTATGTGTGA	CCATTCCAACCATTACTCCCTGA

Sequence-specific primers for NRK-52E cells

	Species	Forward 5'→3'	Reverse 5'→3'
Gapdh	rat	CTGCACCACCAACTGCTTAG	TCAGCTCTGGGATGACCTTG
Col1a1	rat	TCGAGTATGGAAGCGAAGGT	TTGAGGTTGCCAGTCTGTTG
Acta2	rat	ACTGGGACGACATGGAAAAG	GCCACATACATGGCAGGGACATTG

Sequence-specific primers for HK-2 cells

	Species	Forward 5'→3'	Reverse 5'→3'
Gapdh	human	ACCAAATCCGTTGACTCCGAC	CTCCTGTTCGACAGTCAGCC
Col1a1	human	GATTCCCTGGACCTAAAGGTGC	AGCCTCTCCATCTTTGCCAGCA
Acta2	human	ATCACCAACTGGGACGACAT	GGCAACACGAAGCTCATTG

Histological analysis

We fixed the mouse kidneys in 10% formalin neutral buffer solution (Wako) and histologically analyzed them by using the Masson's trichrome method as described previously [1].

In situ hybridization

The kidneys were fixed by perfusion with periodate lysine (0.2 M) and paraformaldehyde (2%) in phosphate-buffered solution. The fixed samples were embedded in an optimum cutting temperature compound (Tissue Tek) and then cryosectioned (5 µm thickness). RNA *in situ* hybridization was performed using the RNAscope 2.5 HD Reagent Kit–BROWN (Advanced Cell Diagnostics, #322300) according to the manufacturer's instructions. We used the Target Probe Mm-*Alox15* (Advanced Cell Diagnostics, #539781) for *Alox15*.

LC-MS/MS-based mediator lipidomics

We conducted LC-MS/MS analysis as described previously [2, 3]. Lipid metabolites were extracted by solid-phase extraction using Monospin C18-AX cartridges (GL Science, Shinjuku, Tokyo, Japan) in the presence of deuterated internal standard: 1 ng of AA-d8, 15-hydroxyeicosatetraenoic acid (HETE)-d8, leukotriene B_4 (LTB_4)-d4, LTD_4-d5, prostaglandin E_2 (PGE_2)-d4, PGB_2-d4, and 9-iso-PGF_{2a}-d4. For LC-MS/MS analysis, a triple-quadrupole linear ion-trap mass spectrometer (5500QTRAP; Sciex,

Framingham, MA, USA) equipped with an ACQUITY UPLC BEH C18 column (1.0 × 150 mm, 1.7-μm particle size; Waters, Milford, MA, USA) was used. MS/MS analyses were conducted in negative ion mode, and lipid metabolites were identified and quantified by multiple reaction monitoring. Calibration curves between 1 and 1000 pg and the LC retention times for each compound were established with synthetic standards.

Statistical analysis

Statistical significance was evaluated using an unpaired t test. For multiplex comparisons, the one-way analysis of variance test with Tukey's test was used. $P < 0.05$ was considered statistically significant. Data are presented as mean ± standard error of the mean (SEM). Statistical analyses were performed using GraphPad Prism 8 (GraphPad Software).

Supplementary References

[1] Kikuchi H, Sasaki E, Nomura N, Mori T, Minamishima YA, Yoshizaki Y, Takahashi N, Furusho T, Arai Y, Mandai S, Yamashita T, Ando F, Maejima Y, Isobe K, Okado T, Rai T, Uchida S, Sohara E. Failure to sense energy depletion may be a novel therapeutic target in chronic kidney disease. Kidney Int. 2019; 95(1):123-37; doi: S0085-2538(18)30635-5 [pii].

[2] Arita M. Mediator lipidomics in acute inflammation and resolution. J Biochem. 2012; 152(4):313-9; doi: mvs092 [pii].

[3] Isobe Y, Itagaki M, Ito Y, Naoe S, Kojima K, Ikeguchi M, Arita M. Comprehensive analysis of the mouse cytochrome P450 family responsible for omega-3 epoxidation of eicosapentaenoic acid. Sci Rep. 2018; 8(1):7954-4; doi: 10.1038/s41598-018-26325-4 [doi].

Supplementary Table

Sample Name	Sham WT (n = 6)		Sham KO (n = 6)		Nx WT (n = 4)		Nx KO (n = 4)		p value
	Ave [pg]	SE	Ave [pg]	SE	Ave [pg]	SE	Ave [pg]	SE	(Nx WT vs Nx KO)
PGE2	29.0	12.8	68.8	46.0	19.3	6.7	42.9	13.3	0.9575
PGD2	10.0	1.5	16.5	5.9	53.4	14.5	163.1	45.1	0.0093
15-keto-PGE2	0.0	0.0	0.0	0.0	0.0	0.0	0.0	0.0	-
15-deoxy-PGJ2	0.0	0.0	0.0	0.0	0.0	0.0	0.0	0.0	-
PGF2a	2.4	0.9	5.8	0.4	1.6	0.9	4.9	1.6	0.1552
6-keto-PGF1a	0.0	0.0	4.8	4.8	134.4	26.1	156.2	62.0	0.9518
TXB2	0.0	0.0	2.9	2.9	16.5	6.1	34.8	10.1	0.1267
12-HHTrE	12.3	2.2	17.5	2.9	38.4	7.9	71.5	20.8	0.127
LTB4	0.0	0.0	0.0	0.0	0.0	0.0	0.0	0.0	-
LTB4-20OH	0.0	0.0	0.0	0.0	0.0	0.0	0.0	0.0	-
LTD4	0.0	0.0	0.0	0.0	0.0	0.0	0.0	0.0	-
HxA3	0.0	0.0	0.0	0.0	0.0	0.0	0.0	0.0	-
HxB3	20.1	20.1	0.0	0.0	20.4	20.4	0.0	0.0	0.815
LXA4	0.0	0.0	0.0	0.0	0.0	0.0	0.0	0.0	-
LXB4	0.0	0.0	0.0	0.0	0.0	0.0	0.0	0.0	-
5-HETE	114.1	17.1	198.4	13.7	91.5	14.4	98.1	19.9	0.9938
5,6-EET	32.9	5.3	48.2	5.9	35.1	8.9	55.7	15.4	0.4308
5,6-DHT	0.0	0.0	0.0	0.0	0.0	0.0	0.0	0.0	-
8-HETE	46.2	2.3	68.8	4.8	39.6	3.3	31.8	5.0	0.6138
9-HETE	17.9	17.9	110.7	35.5	0.0	0.0	0.0	0.0	>0.9999
8,9-EET	0.0	0.0	0.0	0.0	0.0	0.0	0.0	0.0	-
8,9-DHT	0.0	0.0	0.0	0.0	0.0	0.0	0.0	0.0	-
11-HETE	57.1	6.6	99.3	9.9	65.5	3.6	64.6	12.0	>0.9999
12-HETE	141.9	20.8	213.5	39.2	175.8	9.8	183.9	45.1	0.9986
11,12-EET	3.2	1.1	8.1	0.6	2.9	1.7	7.1	0.8	0.0863
11,12-DHT	0.0	0.0	3.5	2.4	2.5	2.5	3.1	3.1	0.998
15-HETE	125.6	18.2	241.7	25.8	177.2	18.8	109.2	9.0	0.2103
14,15-EET	0.0	0.0	0.0	0.0	0.0	0.0	0.0	0.0	-
14,15-DHT	3.6	1.7	9.9	1.5	8.3	1.3	6.0	3.5	0.8897
16-HETE	2.2	1.4	9.4	3.2	7.0	1.6	0.0	0.0	0.2338
17-HETE	0.0	0.0	0.0	0.0	0.0	0.0	0.0	0.0	-
18-HETE	0.0	0.0	0.0	0.0	0.0	0.0	0.0	0.0	-
19-HETE	0.0	0.0	0.0	0.0	0.0	0.0	0.0	0.0	-
20-HETE	0.0	0.0	0.0	0.0	0.0	0.0	0.0	0.0	-
5-oxo-ETE	27.4	9.2	28.2	4.9	13.1	5.9	11.5	2.0	0.9987

12-oxo-ETE	0.0	0.0	0.0	0.0	0.0	0.0	0.0	0.0	-
15-oxo-ETE	25.4	8.6	36.2	4.7	11.0	6.1	10.1	3.9	0.9997
5,15-diHETE	4.9	4.9	7.6	7.6	0.0	0.0	0.0	0.0	>0.9999
8,15-diHETE	0.0	0.0	28.8	9.9	15.5	15.5	15.4	15.4	>0.9999
PGE3	0.0	0.0	0.0	0.0	0.0	0.0	0.0	0.0	-
PGD3	0.0	0.0	0.0	0.0	0.0	0.0	0.0	0.0	-
PGF3a	0.0	0.0	0.0	0.0	0.0	0.0	0.0	0.0	-
6-keto-PGF1a-17delta	0.0	0.0	0.0	0.0	0.0	0.0	0.0	0.0	-
TXB3	0.0	0.0	0.0	0.0	0.0	0.0	0.0	0.0	-
LTB5	0.0	0.0	0.0	0.0	0.0	0.0	0.0	0.0	-
LXA5	0.0	0.0	0.0	0.0	0.0	0.0	0.0	0.0	-
RvE1	0.0	0.0	0.0	0.0	0.0	0.0	0.0	0.0	-
RvE2	0.0	0.0	0.0	0.0	0.0	0.0	0.0	0.0	-
5-HEPE	99.3	11.7	124.8	12.9	120.0	10.8	91.2	20.8	0.5754
5,6-diHETE	0.0	0.0	0.0	0.0	0.0	0.0	0.0	0.0	-
8-HEPE	26.0	6.8	55.5	8.6	57.8	4.1	35.7	8.6	0.2974
9-HEPE	59.4	21.0	119.0	10.9	109.0	20.3	49.2	13.8	0.1706
8,9-EpETE	0.0	0.0	0.0	0.0	0.0	0.0	0.0	0.0	-
8,9-diHETE	0.0	0.0	0.0	0.0	0.0	0.0	0.0	0.0	-
11-HEPE	60.4	7.6	95.0	12.2	101.6	19.7	51.7	11.8	0.0946
12-HEPE	230.3	44.1	399.0	82.8	263.8	34.1	274.7	104.9	0.9997
11,12-EpETE	0.0	0.0	0.0	0.0	0.0	0.0	0.0	0.0	-
11,12-diHETE	0.0	0.0	0.0	0.0	9.4	9.4	11.7	11.7	0.9947
15-HEPE	78.8	8.7	99.4	8.1	219.2	26.7	89.5	25.9	0.0006
14,15-EpETE	0.0	0.0	0.0	0.0	0.0	0.0	0.0	0.0	-
14,15-diHETE	60.0	15.6	50.9	23.4	97.0	12.9	96.5	30.1	-
18-HEPE	136.9	13.3	215.7	17.6	260.8	55.7	118.3	21.7	0.0186
17,18-EpETE	0.0	0.0	0.0	0.0	28.0	28.0	0.0	0.0	0.3891
17,18-diHETE	0.0	0.0	0.0	0.0	0.0	0.0	0.0	0.0	-
19-HEPE	0.0	0.0	0.0	0.0	0.0	0.0	0.0	0.0	-
20-HEPE	0.0	0.0	0.0	0.0	0.0	0.0	0.0	0.0	-
8,18-diHEPE	0.0	0.0	0.0	0.0	0.0	0.0	0.0	0.0	-
11,18-diHEPE-2	5.3	2.4	6.9	4.6	13.1	4.4	0.0	0.0	0.1368
12,18-diHEPE	0.0	0.0	0.0	0.0	54.5	23.0	0.0	0.0	0.0067
17,18-diHEPE-RS	0.0	0.0	0.0	0.0	0.0	0.0	0.0	0.0	-
12hy-17,18-EpETE	0.0	0.0	0.0	0.0	0.0	0.0	0.0	0.0	-
14,15-17,18-diEpETE	0.0	0.0	0.0	0.0	0.0	0.0	0.0	0.0	-
RvD1	0.0	0.0	0.0	0.0	0.0	0.0	0.0	0.0	-
RvD2	0.0	0.0	0.0	0.0	0.0	0.0	0.0	0.0	-

4-HDoHE	396.8	55.8	646.8	74.6	417.3	70.2	360.5	91.7	0.959
7-HDoHE	75.1	4.0	108.9	10.7	107.7	8.9	69.0	13.8	0.0829
8-HDoHE	337.7	43.4	585.3	74.9	335.7	42.2	284.4	47.0	0.9439
7,8-EpDPE	31.4	7.3	38.7	7.5	47.6	7.4	67.4	16.9	0.5772
7,8-diHDoPE	0.0	0.0	0.0	0.0	0.0	0.0	0.0	0.0	-
10-HDoHE	128.8	16.5	256.9	30.7	288.1	54.7	126.7	25.4	0.0246
11-HDoHE	245.9	43.2	488.0	74.5	678.4	146.6	305.5	57.8	0.0443
10,11-EpDPE	25.7	4.5	32.9	4.1	21.0	3.3	40.5	7.3	0.092
10,11-diHDoPE	2.6	2.6	6.1	2.3	4.4	4.4	11.7	9.6	0.7582
13-HDoHE	140.8	17.4	255.2	26.8	388.1	104.6	162.8	31.2	0.0341
14-HDoHE	289.5	25.1	368.6	26.2	673.2	128.3	328.0	57.6	0.0092
13,14-EpDPE	13.4	1.9	19.5	1.8	14.8	3.6	16.8	3.6	0.9631
13,14-diHDoPE	21.2	4.0	23.8	4.7	19.9	5.9	24.9	13.8	0.9663
16-HDoHE	232.1	25.1	369.1	38.3	471.7	106.7	227.0	53.4	0.0497
17-HDoHE	468.5	82.0	513.1	59.0	1253.0	218.2	350.6	88.7	0.0005
16,17-EpDPE	7.3	3.3	15.7	3.5	7.4	4.7	5.0	5.0	0.9789
16,17-diHDoPE	29.9	1.6	32.4	6.8	29.9	5.7	27.5	4.8	0.9912
20-HDoHE	398.9	59.6	700.2	78.1	766.0	206.3	388.5	75.4	0.1414
19,20-EpDPE	220.7	12.4	203.5	20.7	319.2	50.7	218.1	39.2	0.1621
19,20-diHDoPE	93.4	7.9	75.8	12.3	102.4	10.8	105.8	24.3	0.9986
21-HDoHE	116.1	15.0	118.7	12.7	214.9	50.9	124.7	18.9	0.1271
22-HDoHE	0.0	0.0	8.2	5.2	15.9	9.2	12.7	7.4	0.9825
17-oxo-DoHE	27.8	12.7	56.2	16.7	99.5	25.4	67.6	22.6	0.6958
4,14-diHDoHE	0.0	0.0	0.0	0.0	0.0	0.0	0.0	0.0	-
7,14-diHDoHE	0.0	0.0	0.0	0.0	0.0	0.0	0.0	0.0	-
7,17-diHDoHE	0.0	0.0	0.0	0.0	0.0	0.0	0.0	0.0	-
10,17-diHDoHE	0.0	0.0	5.6	5.6	21.9	8.0	0.0	0.0	0.0381
14,20-diHDoHE	0.0	0.0	31.1	21.5	104.6	28.1	0.0	0.0	0.0067
16:0 Palmitic Acid	91902.6	10170.4	82738.2	5263.1	191861.6	22821.2	143310.6	27204.8	0.2328
16:1 Palmitoleic Acid	15658.9	3956.6	9630.7	1623.5	81329.7	17316.3	48814.0	14128.6	0.1459
18:0 Stearic Acid	45670.5	4471.5	48051.3	3748.8	58167.9	8210.8	55004.4	6115.5	0.9814
18:1 Oleic Acid(n9)	62253.5	9160.4	51236.5	4697.2	149180.4	18033.3	118453.8	18800.9	0.3889
18:2 Linoleic Acid	206766.9	25926.2	177144.1	15532.9	586290.5	71210.7	415077.8	84585.1	0.1346
18:3 a-Linolenic Acid(n3)	17294.0	3733.9	14980.1	1876.3	85228.7	10241.5	50273.1	16420.4	0.0571
18:3 g-Linolenic Acid(n6)	1191.7	133.2	1089.2	100.4	2957.9	282.8	1996.5	589.1	0.1579
18:4 Stearidonic Acid	1169.9	178.1	1211.4	142.2	5502.3	738.9	3694.6	1387.2	0.2957
20:3 DGLA(n6)	9794.6	349.6	8797.8	791.8	22167.8	3136.0	13040.0	2866.7	0.0195
20:3 Mead Acid(n9)	222.7	24.9	224.1	32.7	359.4	68.6	314.7	29.7	0.8806
20:4 ETA(n3)	1098.3	134.0	1090.4	78.2	4297.5	752.6	2587.0	812.5	0.1049

20:4 Arachidonic Acid(n6)	47987.9	4501.0	49800.5	4022.6	45471.4	8398.2	42217.4	7657.4	0.9837
20:5 EPA	30513.0	1937.7	32268.5	2776.5	33619.7	8544.9	26004.7	7873.1	0.7669
22:4 Adrenic Acid	1777.1	79.2	2004.2	237.4	3741.6	363.5	2732.3	296.3	0.0696
22:5 DPA(n3)	24774.2	1133.0	25339.4	2371.4	31422.8	2516.3	23383.2	3366.1	0.1596
22:5 Osbond Acid(n6)	1547.1	183.8	1380.3	209.9	3673.0	506.2	2556.3	398.4	0.1312
22:6 DHA	222989.5	11473.8	219463.9	17716.7	207464.4	21392.0	168665.0	23335.3	0.5304

Supplementary Table 1

List of all the lipid metabolites in sham and 5/6 Nx kidneys which were analyzed with the mediator lipidomics. By mediator lipidomics, the above fatty acid metabolites were detected in the kidney tissue (30 mg). The P values in the table were obtained by comparing *Alox15*$^{+/+}$ and *Alox15*$^{-/-}$ mice under 5/6 Nx condition. The number of samples is as follows: sham (WT), n = 6, sham (KO), n = 6, Nx (WT), n = 4, Nx (KO), n = 4. One-way analysis of variance was followed by Tukey’s multiple comparisons test.
